# Rhabdomyolysis: An Unusual Presentation of *Mycoplasma pneumoniae* Infection in an Adult—A Case Report and Literature Review

**DOI:** 10.1155/2018/6897975

**Published:** 2018-06-21

**Authors:** Jaspreet Kaler, Osama Mukhtar, Bilal Khan, Binav Shrestha, Ravinder Kaler, Brandon Ting, Mazin Khalid

**Affiliations:** ^1^Department of Medicine, Interfaith Medical Center, Brooklyn, NY, USA; ^2^Caribbean Medical University, Curacao, Netherlands; ^3^Avalon University School of Medicine, Curacao, Netherlands

## Abstract

*Mycoplasma pneumoniae* is a common cause of community-acquired pneumonia, and many extrapulmonary manifestations have been described, but rhabdomyolysis is infrequently reported in adults. Of the few cases that have been reported in adults, it was almost exclusively seen when pneumonia was present. We report a case of a 30-year-old male who came in with complaints of fever and myalgia for three days. Immunoglobulin M antibodies for *Mycoplasma pneumoniae* were positive and trending up, despite having no radiographic evidence of pneumonia on chest X-ray or CT scan. He was treated successfully with levofloxacin and intravenous hydration. Later, his condition was clinically and biochemically improved, and he was discharged. Our patient did not present with typical respiratory tract symptoms of a mycoplasma infection. In addition, there was an absence of pneumonia on imaging, suggesting that rhabdomyolysis secondary to mycoplasma might be underdiagnosed and go untreated in the setting of low clinical suspicion. Upon review of the literature, there is only one other case of mycoplasma infection where rhabdomyolysis occurred in the absence of pneumonia. However, the degree of rhabdomyolysis in our case was much more severe. Although rare, when faced with rhabdomyolysis, *Mycoplasma pneumoniae* should be kept as a differential diagnosis even in the absence of pneumonia on radiological imaging.

## 1. Introduction

*Mycoplasma pneumoniae* is a rod-shaped bacterium, which affects 1% of the United States (US) population annually [1]. It is one of the most common causes of atypical pneumonia in the US, and it is transmitted from person to person via respiratory droplets. This disease has an incubation period of 2-3 weeks and can affect people of any age group but is most commonly found in school-aged children and military and college students. When infected, patients can present with clinical symptoms or be completely asymptomatic. Typical symptoms include headache, malaise, fever, chills, and cough. In fact, 75–100% of infected patients have an intractable nonproductive cough and 3–10% of infected patients develop pneumonia [2]. Extrapulmonary disease occurs in less than 5–10% of patients infected with mycoplasma [3]. This can include hematological, dermatological, gastrointestinal, neurological, and musculoskeletal manifestations. Of the several musculoskeletal manifestations, rhabdomyolysis has rarely been seen in *Mycoplasma pneumoniae*. In the cases that have been reported, rhabdomyolysis usually occurs in the pediatric population. We present an unusual case of *Mycoplasma pneumoniae* infection presenting with rhabdomyolysis in an adult patient where no radiographic evidence of pneumonia was detected.

## 2. Case Presentation

A 30-year-old male, with no significant past medical history, came to the emergency department with complaints of fever and chills for a duration of 3 days. He also complained of myalgia and abdominal pain for the same duration. He reported diffuse abdominal pain, which was 6/10 in severity, nonradiating, and not related to food. He also reported 2 episodes of diarrhea without any blood or mucous, brown in color. He denied cough, shortness of breath, chest pain, night sweats, weight loss, history of trauma, extreme exercise, or any history of travel.

His abdominal examination was significant for abdominal tenderness in all four quadrants, but was soft and nondistended and bowel sounds were present. The rest of his examination was unremarkable. Triage vitals were significant for a temperature of 103.3°F, heart rate of 88 beats per minute, respiratory rate of 18 breaths per minute, blood pressure of 109/49, and pulse oximetry of 99% on room air.

Admission laboratory workup revealed leukocyte count of 6.1 × 10^3^ *µ*L (reference range 4.5–11.0 × 10^3^) with 72% neutrophils, hemoglobin of 16.2 g/dL (reference range 13.0–17.0), hematocrit of 48.2% (reference range 39–53), platelet count of 149 × 10^3^ *µ*L (reference range 130–400 × 10^3^), ESR of 4 mm/hr (reference range 0–20), BUN of 34 mg/dL (reference range 8–20), creatinine of 1.8 mg/dL (reference range 0.4–1.3), potassium of 4.7 mmol/L (reference range 3.6–5.1), phosphorous of 3.0 mg/dL (reference range 2.4–4.7), aspartate aminotransferase of 1,146 IU/L (reference range 15–41), alanine aminotransferase of 243 IU/L (reference range 17–63), and creatinine kinase of 39,125 IU/L (reference range 38–398). Urinalysis was significant for large blood, and urine red blood cells were 0–2.

**Figure 1:**
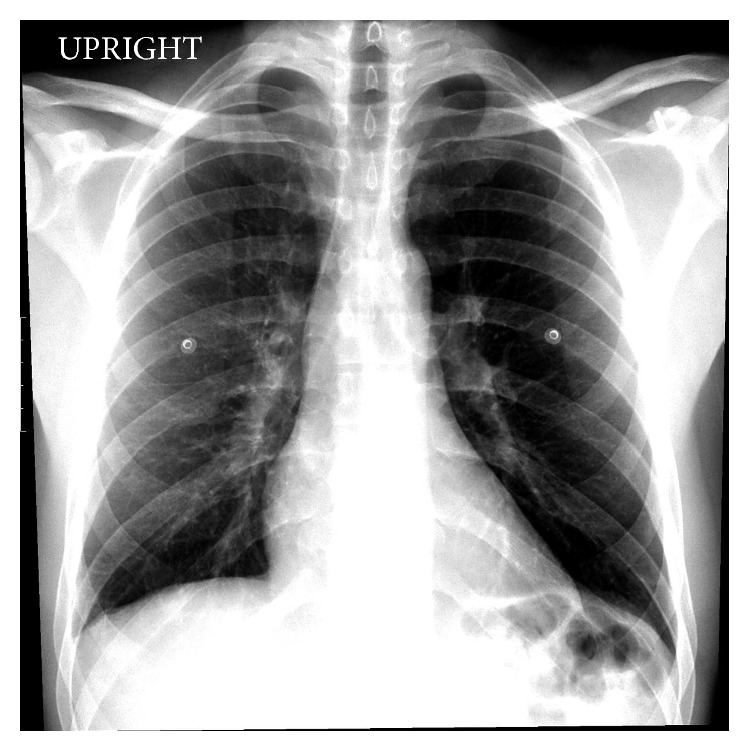
Chest X-ray showing no evidence of acute lung pathology.

In view of the high-grade fever, we attempted to find an infectious source to explain his history and presentation. Chest X-ray (Figure 1) and CT scan were obtained and showed no signs of pneumonia. Urinalysis and influenza rapid antigen test were performed and did not reveal any abnormalities. CT of the abdomen and pelvis was obtained and did not reveal any focal sources. Stool culture with ova and parasites, blood cultures, urinary toxicology including synthetic THC (K2), HIV, herpes simplex virus, hepatitis panel, *Mycoplasma pneumoniae* IgM antibody, urine Legionella antigen, QuantiFERON® test for tuberculosis, and TSH were all performed as well. All the test results were negative except immunoglobulin M antibodies for *Mycoplasma pneumoniae* that were detected by enzyme-linked immunosorbent assay. The antibodies were initially 781 U/mL, which is considered a low positive (reference range 770–950).

**Figure 2:**
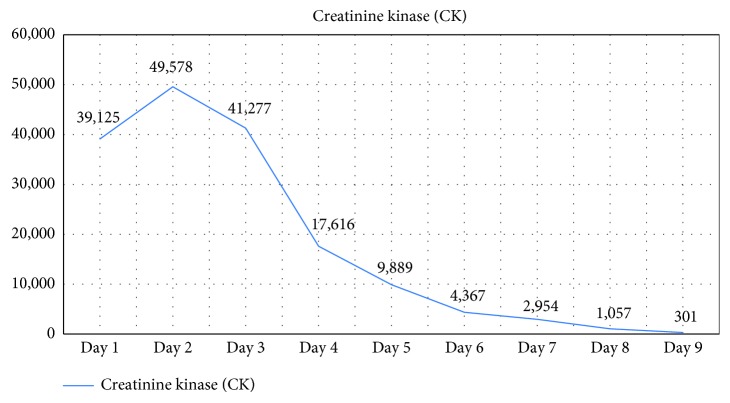
Creatinine kinase trend during hospital stay.

Our patient was empirically started on levofloxacin and metronidazole for possible gastroenteritis initially while culture and laboratory results were pending. He was managed for his acute renal failure and rhabdomyolysis with aggressive parenteral hydration. However, he continued to spike fevers during days 1–4 of the admission, but his diarrhea, abdominal pain, and myalgia resolved by day 3. One week later, mycoplasma antibody was recorded again and it was 976 U/ml, which is considered a true positive (reference range 950+). The fact that the antibody titer was trending up was clinically significant for a recent *Mycoplasma pneumoniae* infection. After mycoplasma was found to be positive, metronidazole was discontinued and he was continued with levofloxacin. His creatinine, liver function tests, and CK trended down to normal reference ranges (Figure 2), and he was discharged on day 9 with planned outpatient follow-up.

## 3. Discussion

The pathophysiology of rhabdomyolysis is based upon muscle necrosis, resulting in a release of intracellular components. This results in an increase of creatinine kinase and myoglobin in urine. Clinically, rhabdomyolysis can be asymptomatic or it can present with myalgia and lead to renal failure with electrolyte derangements. There are a variety of triggers that result in rhabdomyolysis, which includes trauma, extreme exertion, metabolic myopathy, thermal extremes, hypothyroidism, medications, drugs (alcohol, heroin, cocaine, amphetamine, methadone, D-lysergic acid diethylamide), electrolyte disorders (hypokalemia, hypophosphatemia), and certain infections. Infections account for less than 5% of all rhabdomyolysis causes [4]. Infectious causes include influenza A and B, coxsackievirus, Epstein–Barr virus, herpes simplex, parainfluenza, adenovirus, echovirus, enterovirus, human immunodeficiency virus, cytomegalovirus, varicella-zoster, *Mycoplasma pneumoniae*, *Legionella pneumophila*, tularemia, *Streptococcus pneumoniae*, *Salmonella*, *E. coli*, leptospirosis, *Coxiella burnetii*, *Staphylococcus*, ehrlichiosis, falciparum malaria, *Clostridium perfringens,* and *Chlamydia psittaci* [5–19]. This patient's history, clinical data, rising titers of mycoplasma antibodies, and his clinical response to levofloxacin are all compatible with *Mycoplasma pneumoniae* being the trigger for rhabdomyolysis.

Rhabdomyolysis triggered by *Mycoplasma pneumoniae* is a rare extrapulmonary manifestation in adults. Our case is unique since there was no radiographic evidence of pneumonia. Review of literature has shown that the earliest case of rhabdomyolysis in an adult with *Mycoplasma pneumoniae* was reported as early as 1979 [20]. To our knowledge, 7 cases of *Mycoplasma pneumoniae* with rhabdomyolysis have been described in adults since then (Table 1) [20–26]. Of the reported cases, Decaux et al. reported the one other case, besides ours, where a 60-year-old male presented with rhabdomyolysis and acute encephalitis secondary to *Mycoplasma pneumoniae* but without pneumonia [21]. However, the degree of rhabdomyolysis in our case was much more severe. Our case had a CK of 49,578 at its height, compared to the Decaux et al.'s case that had a CK of 2,900 [21]. The reported cases of rhabdomyolysis with mycoplasma (Table 1) revealed that the severity of rhabdomyolysis varies from case to case. Furthermore, additional extrapulmonary manifestations, besides rhabdomyolysis, were present in some of the cases as well.

The exact mechanism for rhabdomyolysis in *Mycoplasma pneumoniae* is unclear, but the cases reported suggest a definite association. Mechanisms that have been proposed to cause extrapulmonary manifestations of mycoplasma include direct inflammatory cytokines, indirect autoimmunity or immune complexes, and occlusion of blood vessels causing damage [8, 27]. It has been suggested that TNF-alpha may be the cause of rhabdomyolysis in mycoplasma although this has not been confirmed yet [28].

The diagnosis of rhabdomyolysis is usually suggested by myalgia or a urinalysis that tests positive for blood in the absence of red blood cells on microscopy. Rhabdomyolysis is formally diagnosed with a CK level 5 times the upper limit of normal and is usually managed with aggressive intravenous hydration to prevent myoglobin-induced renal failure. In general, 50% of patients who develop rhabdomyolysis also develop renal failure [4, 9]. Diagnosis of *Mycoplasma pneumoniae* infection, on the other hand, is more difficult to suspect due to the absence of radiographic evidence of pneumonia in some cases. In addition, these patients may not even present with respiratory tract symptoms as seen with our patient. In this case, the patient presented with fever, myalgia, and abdominal complaints, which are not pathognomonic of most mycoplasma cases. The most accurate test to confirm the diagnosis of *Mycoplasma pneumoniae* infection is a throat culture. However, this is not typically done, as it takes 2-3 weeks to obtain the results. Because of the element of time, polymerase chain reaction (PCR) is typically done when it is available, but most often serology for mycoplasma immunoglobulin M is performed. Treatment of *Mycoplasma pneumoniae* infection includes antibiotics such as macrolides, fluoroquinolones, or doxycycline, which is what our patient responded to.

## 4. Conclusion

Traditionally, *Mycoplasma pneumoniae* is a common cause of atypical community-acquired pneumonia, and many extrapulmonary manifestations have been described. Rhabdomyolysis, specifically, is rarely reported in adults with mycoplasma, especially when no radiographic evidence of pneumonia is present. In this case, there were no typical suggestive complaints for mycoplasma infection, suggesting that rhabdomyolysis secondary to mycoplasma might be underdiagnosed and go untreated. Even though rhabdomyolysis is a rare extrapulmonary manifestation of *Mycoplasma pneumoniae* infection, it should be kept as a differential diagnosis when faced with rhabdomyolysis of unknown etiology despite the absence of pneumonia.

## Figures and Tables

**Table 1:** Reported cases of rhabdomyolysis in adults with *Mycoplasma pneumoniae* infection.

Reported cases	Date reported	Age/gender	Pneumonia	Creatinine kinase	Other extrapulmonary symptoms
Rothstein and Kenney [20]	1979	28/F	Present	>40,000	Neurological
Decaux et al. [21]	1980	60/M	Absent	2,900	Neurological
Daxbock et al. [22]	2002	55/M	Present	792	Dermatological
Gupta et al. [23]	2005	25/F	Present	77,700	Neurological
Khan et al. [24]	2013	37/M	Present	14,220	—
Sertogullarindan et al. [25]	2013	25/F	Present	51,226	—
Gosselt et al. [26]	2017	47/M	Present	25,422	Dermatological
Current case	2018	30/M	Absent	49,478	Gastrointestinal
